# Rectus diastasis repair with and without mesh at 1 year: randomized clinical trial

**DOI:** 10.1093/bjs/znaf231

**Published:** 2025-11-25

**Authors:** Reetta Tuominen, Julia Saxen, Tiina Jahkola, Jani Mikkonen, Jari Arokoski, Hannu Luomajoki, Jaana Vironen

**Affiliations:** Department of Plastic Surgery, Helsinki University Hospital and University of Helsinki, Helsinki, Finland; Department of Plastic Surgery, Helsinki University Hospital and University of Helsinki, Helsinki, Finland; Department of Plastic Surgery, Helsinki University Hospital and University of Helsinki, Helsinki, Finland; Department of Surgery (incl. Physiatry), Institute of Clinical Medicine, University of Eastern Finland, Kuopio, Finland; Department of Physical and Rehabilitation Medicine, Helsinki University Hospital and University of Helsinki, Helsinki, Finland; ZHAW School of Health Professions, Zurich University of Applied Sciences, Winterthur, Switzerland; Abdominal Centre, Helsinki University Hospital and University of Helsinki, Jorvi, Espoo, Finland

## Abstract

**Background:**

Rectus diastasis (RD) can cause functional impairment and pain. Surgical approaches vary regarding mesh use for linea alba restoration. The aim of this RCT was to compare plication supported by mesh (PSUM) with suture plication in patients with symptomatic postpartum RD.

**Methods:**

This single-centre superiority double-blind RCT was conducted in Finland from April 2018 to November 2022. The pre-specified primary outcome was RD recurrence at 1 year (inter-rectus distance (IRD) >20 mm) and a post-hoc primary outcome was absolute reduction in IRD. Secondary outcomes included health-related quality of life (HRQoL), low back disability (Oswestry Disability Index (ODI)), motor control, and complications. The date of the last follow-up was 30 November 2022.

**Results:**

In total, 86 normal-weight women (mean age of 38.7 (range 25–52) years) were randomized, with 84 patients (98%) available for follow-up (44 in the PSUM group and 40 in the suture plication group). At 1 year, there was no difference in RD recurrence between the PSUM group and the suture plication group (2 of 44 (5%) *versus* 2 of 40 (5%) respectively; *P* = 0.922). IRD reduction was greater in the PSUM group (52 (95% c.i. 48 to 56) mm) than in the suture plication group (44 (95% c.i. 40 to 47) mm) (*P* < 0.002). HRQoL, ODI, and enhanced performance test results all improved in both groups compared with baseline (*P* = 0.000–0.039). There was no difference in the complication rate between the PSUM group and the suture plication group (17 of 44 (39%) *versus* 13 of 40 (33%) respectively; *P* = 0.558).

**Conclusion:**

At 1 year, both PSUM and suture plication result in stable outcomes, with no difference in the RD recurrence rate, but the study was limited by both a small number of patients and a lower than anticipated overall RD recurrence rate. For the whole patient cohort, surgery was associated with improved motor control, low back function, and HRQoL compared with baseline.

**Registration number:**

NCT03509376 (http://www.clinicaltrials.gov).

## Teaser

This RCT compared plication supported by mesh (PSUM) with suture plication for rectus diastasis (RD) repair in 84 women. PSUM resulted in greater inter-rectus distance (IRD) reduction, while both techniques improved health-related quality of life, motor control, and low back function. PSUM enhances IRD reduction and provides added linea alba plication stability, with the complication rate similar between groups.

## Introduction

Wide rectus diastasis (RD) can lead to disabilities, low back pain, core instability, aesthetic concerns, and reduced quality of life (QoL)^1,2^. Core stability exercises targeting the abdominal and pelvic floor muscles are considered the first-line treatment for RD, with surgical intervention reserved as a secondary option^1,3^. However, studies have shown that, while these exercises can lead to improvements, their effectiveness in completely resolving RD is often modest^4^. Operative management has increased in popularity during the past decade and has been shown to reduce back pain and improve QoL^5,6^. While surgical repair has been suggested to be more effective for selected patients, neither physiotherapy nor surgery guarantees complete resolution, as noted in the European Hernia Society (EHS) guidelines^1,7^.

RD is often linked to midline hernias due to a stretched, thin linea alba^8^. General, abdominal, and plastic and reconstructive surgeons encounter RD patients, with treatment strategies differing by specialty^5,9,10^. A variety of surgical techniques have been described, such as linea alba plication with or without mesh augmentation, via an open or laparoendoscopic approach^9,11^. However, there is no consensus on the optimal method. Evidence suggests low recurrence rates after plication, but imaging validation and long-term outcome results are limited^5^. Plication supported by mesh (PSUM) is a technique in which a mesh is positioned in the plane between the reapproximated rectus muscles during abdominoplasty (*Fig. S1*)^12^. A preliminary PSUM technique study reported a good subjective improvement in body balance after surgery, with a low complication rate (total complication rate of 14% (5 of 37)) and high patient satisfaction. However, gaps remain in the understanding of the long-term efficacy of PSUM and the outcomes of PSUM compared with those of other surgical techniques, which this trial aims to address.

The aim of this study was to compare PSUM with non-absorbable suture plication without mesh. The primary aim of this superiority double-blind RCT was to assess the risk of RD relapse 1 year after operative treatment, with relapse defined as an inter-rectus distance (IRD) >20 mm.

## Methods

### Trial design

This superiority double-blind RCT was conducted at the Department of Plastic and Reconstructive Surgery and the Abdominal Centre at Helsinki University Hospital, Jorvi, Espoo, Finland. The study protocol was approved by the Regional Ethics Committee of Helsinki University Hospital (Dnr HUS/3094/2017) and registered at ClinicalTrials.gov (NCT03509376). Written informed consent was obtained from all participants. The study followed the CONSORT 2010 guidelines^13^.

### Participants and follow-up

Symptomatic RD was defined as low back pain, inability to resume previous physical activities, or impaired core stability. Inclusion criteria were an IRD >30 mm, symptomatic RD, BMI <28 kg/m^2^, age >18 years, and at least 1 year since last pregnancy or breastfeeding, with no intention of future pregnancy. Exclusion criteria were current smoking and use of immunosuppressive medication. Preoperative assessments included clinical evaluation of hernia presence and size, and manual measurement of the IRD in the outpatient clinic. Eligibility required an IRD >30 mm. Baseline IRD was confirmed intraoperatively using a caliper and postoperative measurements were obtained using ultrasonography. The primary outcome was defined to be assessed at 1 year, but the original study protocol allowed a median-term follow-up over 3 years. Participants were evaluated at three time points: baseline, after 3–6 months of preoperative core stability exercises, and 1 year after surgery.

### Interventions

All participants received a written exercise programme at enrolment, consisting of three sessions per week for at least 3 months. It targeted pelvic floor activation, transversus abdominis engagement, oblique and rectus abdominis activation, and proper alignment during exercise.

Two surgical approaches were compared. In the suture plication arm, RD repair was performed using monofilament, non-absorbable running sutures alone (Prolene^®^ 2-0). In the second arm, the same plication was supported by mesh (PSUM technique), in which a narrow (20 mm) partially absorbable self-gripping mesh strip (Progrip^®^, Covidien) was positioned within a tunnel created by the linea alba plication, with alternate sutures passing through the mesh (*Fig. S1*)^12^. Mesh placement was adapted at the umbilical stalk. Standard abdominoplasty was performed in both groups, including flap elevation, umbilical repositioning, excision of redundant tissue, and layered closure. Hernia sacs, if present, were reduced or resected.

### Outcome measures

The pre-specified primary outcome was RD recurrence at 1 year, defined as IRD >20 mm, assessed using ultrasonography. A post-hoc primary outcome was absolute reduction in IRD. The IRD measurements were taken 30 mm above the umbilicus^14^. Secondary outcomes included health-related quality of life (HRQoL) (measured using RAND-36)^15,16^, low back disability (measured using the Oswestry Disability Index (ODI))^17^, motor control function, urinary symptoms, and postoperative complications (classified in accordance with Clavien–Dindo)^18^. The comprehensive patient questionnaire also recorded sociodemographic data, obstetric history, physical activity, abdominal wall satisfaction, and urinary symptoms (4 items, score 0–8).

Motor control was evaluated using four validated Luomajoki tests: the waiter’s bow test (flexion control), the pelvic tilt test (extension control), the one-leg stance test (lateral/rotational control), and the compression active straight leg raise (ASLR) test^19,20^. The simplified sit-up test (repetitions with arms crossed and elbows touching knees) was added as a measure of core strength. Tests were video recorded and assessed by a blinded expert.

### Randomization and blinding

Randomization was performed electronically using an electronic case report form (eCRF) immediately before surgery, after completion of the preoperative exercise interval. The eCRF used permuted blocks of fixed size 10 to assign participants in a 1:1 ratio, without stratification by centre. The midline repair for all participants was performed by a single experienced hernia surgeon (J.V.) and the study surgeon did not participate in the follow-up. The study surgeon, radiologist, study physiotherapist, and video analyst were blinded to the study group. The patient records did not directly state the randomized procedure, but, if needed, the information was available from the researchers.

### Statistical methods

Sample size was based on an expected recurrence rate of 30% in the suture plication group *versus* 5% in the PSUM group at 1 year^21^. With 80% power at α = 0.05, 46 participants per arm were required. All statistical analyses were conducted using NCSS 12 Statistical Software by a professional statistician. The *P* value indicating significance was set at 0.050 (two-sided). Continuous outcomes (HRQoL scores, sit-up test, and IRD) were compared between groups using an independent-sample *t* test when normally distributed and the Mann–Whitney *U* test otherwise. Within-group changes over time were assessed using a paired *t* test or the Wilcoxon signed rank test, as appropriate. Dichotomous functional outcomes were compared between groups using Fisher’s exact test, while paired binary outcomes were evaluated using the McNemar mid-*P* test. Complication rates were compared using Pearson’s chi-squared test.

## Results

### Participant flow and follow-up

**Fig. 1:**
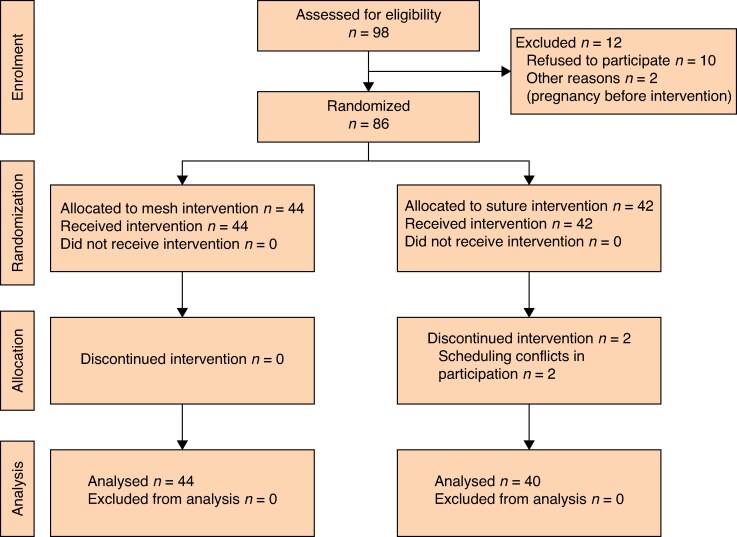
CONSORT flow chart

Patients with symptomatic RD were recruited between 1 April 2018 and 15 November 2021. The trial was terminated due to prolonged study duration due to COVID-19 pandemic-related delays, which also delayed the intended 1 year follow-up to a mean of 15.1 months (PSUM 11.5–44.5 months and suture plication 11.2–33.8 months). The study flow chart is presented in *Fig. 1*. Of the 98 candidates assessed, 10 declined randomization or later opted out of treatment and 2 were excluded due to pregnancies before surgery, leaving 86 postpartum women, who were randomized to undergo either PSUM or suture plication. There were two dropouts after randomization, both from the suture group, resulting in 84 participants available for follow-up (44 in the PSUM group and 40 in the suture plication group). The follow-up ranged from 11.5 to 44.5 months in the PSUM group and from 11.2 to 33.8 months in the suture plication group.

### Baseline characteristics

The baseline demographics and clinical characteristics of the groups are presented in *Table 1* The groups were similar in age, BMI, and number of pregnancies, with no statistically significant differences. The manually measured preoperative IRD was statistically significantly wider in the PSUM group compared with the suture plication group (mean(s.d.) of 61(12) mm *versus* 54(11) mm respectively; *P* = 0.013). Hernias were more frequent in the PSUM group compared with the suture plication group, occurring in 10 of 44 patients (23%; mean size: 10 (range 5–20) mm) and 5 of 40 patients (13%; mean size: 12 (range 5–10) mm) respectively (*P* = 0.261).

**Table 1:** Patient demographics and group comparison (mesh *versus* suture)

	PSUM group (*n* = 44)	Suture plication group (*n* = 40)	*P*
Age (years)	37.8(5.3)	39.6(5.6)	0.134
BMI (kg/m^2^)	23.7(2.1)	23.6(2.7)	0.887
Number of pregnancies	2.4(0.8)	2.4(0.8)	0.841
Preoperative IRD (mm)*	61(12)	54(11)	0.013
Perioperative IRD (mm)†	54(12)	49(15)	0.016
Number of patients with a hernia	10	5	
Hernia size (mm), mean	10	12	

Values are mean(s.d.) unless otherwise indicated. *Preoperative IRD manually measured during consultation. †Perioperative IRD measured using a caliper. PSUM, plication supported by mesh; IRD, inter-rectus distance.

### Primary outcomes: recurrence rate and IRD changes

The recurrence rate was 5% (2 of 44) in the PSUM group and 5% (2 of 40) in the suture plication group (*P* = 0.922). In the PSUM group, the two patients with recurrent RD had postoperative IRDs of 25 and 28 mm. Both patients in this group requested reoperation due to aesthetic concerns and they underwent 70 mm total plication and 60 mm total plication respectively. In the suture plication group, the two patients with recurrent RD had postoperative IRDs of 24 and 30 mm, with the latter patient undergoing reoperation due to dissatisfaction with a persistent abdominal protrusion; 60 mm total plication in the horizontal plane was performed. No hernia recurrences were observed in either group.

There was no difference in the mean postoperative IRD between the PSUM group and the suture plication group (10 (95% c.i. 8 to 12) mm *versus* 12 (95% c.i. 10 to 14) mm respectively; *P* = 0.066). The reduction in IRD was statistically significantly greater after PSUM compared with suture plication (mean IRD decrease: 52 (95% c.i. 48 to 56) mm *versus* 44 (95% c.i. 40 to 47) mm respectively; *P* < 0.002).

### Secondary outcomes: functional and patient-reported outcomes

**Fig. 2:**
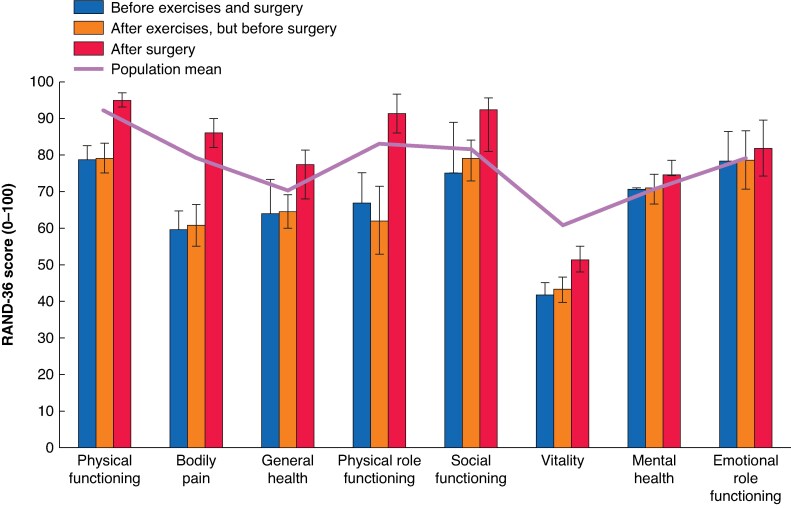
RAND-36 dimensions: outcomes with 95% confidence intervals before exercises and surgery, after exercises, but before surgery, and 1 year after surgery compared with Finnish population norms (Aalto *et al*.^12^)

The HRQoL scores measured using RAND-36 showed statistically significant improvements in most domains between baseline and 1 year follow-up for the whole patient cohort (*Fig. 2* and *Table 2*). There were no statistically significant improvements between baseline and after the preoperative exercise interval. Statistically significant improvements (*P* < 0.05) were observed in all domains except for mental health and emotional role functioning between baseline and 1 year follow-up. The ODI score showed a statistically significant reduction after surgery in all patients, indicating improved low back function. The preoperative mean ODI score for the whole patient cohort was 16% (95% c.i. 13% to 18%), which decreased to 4% (95% c.i. 3% to 5%) after surgery (*P* < 0.000). In all patients, urinary symptoms showed statistically significant improvement after surgery. Among participants with preoperative urinary symptoms (baseline score ≥1; 60 patients), the mean score decreased from 1.7 (95% c.i. 1.4 to 2.0) to 1.1 (95% c.i. 0.8 to 1.4) after surgery (*P* = 0.000). Similarly, in a subgroup with more severe symptoms (baseline score ≥2; 20 patients), the mean score improved from 3.1 (95% c.i. 2.5 to 3.7) to 2.0 (95% c.i. 1.0 to 2.9) after surgery (*P* = 0.009). There were minor differences in the results in some parameters of the RAND-36 between the two groups. Preoperatively, vitality was higher in the mesh (PSUM) group than in the suture group (45.9 *versus* 37.4; *P* = 0.018). Postoperatively, physical functioning (96.0 *versus* 93.4; *P* = 0.022) and social functioning (95.5 *versus* 88.2; *P* = 0.006) favored the mesh group. Preoperative ODI was lower in the mesh group (13.2% *versus* 18.4%; *P* = 0.044). Urinary symptoms did not differ between the PSUM and suture plication groups at either time point (*Tables S1*, *S2*).

**Table 2:** Mean HRQoL scores for the whole patient cohort with *P* values for changes between time points

RAND-36 domains	Before exercise and surgery (baseline)	After exercise, but before surgery	After surgery	*P* value for change between baseline and after exercise, but before surgery	*P* value for change between baseline and after surgery
Physical functioning	78.7	79.2	95.1	0.401	0.000
Bodily pain	59.8	60.8	86.0	0.740	0.000
General health	64.0	64.5	77.3	0.923	0.000
Physical role functioning	66.9	62.1	91.3	0.049	0.000
Social functioning	75.3	78.5	92.2	0.425	0.000
Vitality	41.7	43.2	51.5	0.195	0.000
Mental health	70.7	70.6	74.7	0.664	0.100
Emotional role functioning	78.5	78.6	81.9	0.573	0.443

HRQoL, health-related quality of life.

**Fig. 3:**
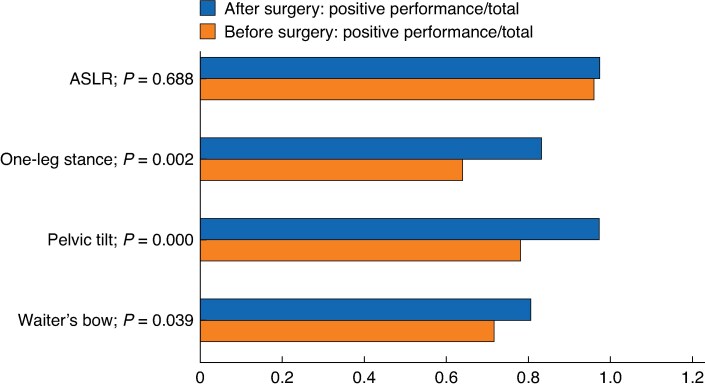
Motor control tests: expert-reviewed performance proportions ASLR, active straight leg raise.

Assessments of core stability performance revealed statistically significant postoperative improvements in the waiter’s bow, pelvic tilt, and one-leg stance tests (*P* = 0.000–0.039), but there was no change in the ASLR test (*P* = 0.688) (*Fig. 3*). Performance in the sit-up test showed statistically significant postoperative improvement; at baseline the mean number of repetitions was 0.9 (95% c.i. 0.3 to 1.5) compared with 3.7 (95% c.i. 2.6 to 4.7) at 1 year follow-up (*P* = 0.000). There were no differences in motor control and sit-up tests between the PSUM group and the suture plication group before or after surgery (*Table S3*).

### Complications

Complications are summarized in detail in *Table 3*. In the PSUM group, 17 of 44 patients (39%) experienced complications, including 3 patients (7%) requiring oral antibiotics (Clavien–Dindo grade IIa). In the suture plication group, 13 of 40 patients (33%) experienced complications, including 1 patient requiring oral antibiotics, and 1 patient with haematoma evacuation under general anaesthesia (5%; Clavien–Dindo grade IIa–III). There was no difference in the overall complication rate between the groups (*P* = 0.558). Additionally, one patient in the PSUM group contracted SARS-CoV-2 infection at 40 days after surgery, which was subsequently complicated by the development of a pulmonary embolism at 6 weeks after surgery.

**Table 3:** Clavien–Dindo classification of complications for the study groups within 30 days of operation

Complications	PSUM group (*n* = 44)	Suture plication group (*n* = 40)
**Clavien–Dindo grade Ia**		
Local haematoma/bruising	1	5
**Clavien–Dindo grade Ib**		
Wound dehiscence	6	2
Local antibiotics	1	1
Suture fistula	3	2
**Clavien–Dindo grade Ic**		
Bedside haematoma/seroma puncture	2	1
Umbilical necrosis	1	
**Clavien–Dindo grade IIa**		
Oral antibiotics	3	1
**Clavien–Dindo grade III**		
Haematoma evacuation under general anaesthesia		1
Total	17	13

## Discussion

This RCT comparing PSUM with suture plication alone for the repair of postpartum RD in normal-weight women showed no difference in the RD recurrence rate (2 of 44 *versus* 2 of 40 respectively) at 1 year. A greater reduction in IRD was observed in the PSUM group. The PSUM group had a mean IRD reduction 6 mm greater than the suture plication group, which may reflect the larger baseline IRD in that group. Both groups achieved postoperative IRD values near accepted thresholds. Both groups experienced marked postoperative improvements in HRQoL, ODI, physical performance, core stability tests, and urinary symptoms compared with baseline.

While the definition of RD relapse varies between 20 and 30 mm, this study defined relapse as an IRD >20 mm^1,5,21^. Based on clinical experience, an IRD of 30 mm after surgery is not considered satisfactory. The EHS defines RD as a separation >20 mm^9^ and a postoperative IRD greater than this may indicate incomplete repair. A persistent wide IRD may result from unaddressed lateral fascial laxity during surgery^22^. Findings at reoperation in the present study, which revealed the need for more extensive fascial plication, support a surgical approach that addresses the full width of anterior abdominal wall laxity, rather than merely reapproximating the medial rectus borders^23^.

In patients with symptomatic RD, laxity and redundancy of the abdominal fascia contribute to core instability and lower back pain^2,5,24^. These individuals benefit from restoration of functional tension. The postoperative improvements in HRQoL and ODI in this study align with prior research^5,21^ . The clinically relevant ODI score improvement highlights the value of RD repair in symptomatic patients in alleviating low back pain and improving function^17^. Clinical assessments of core stability, such as the waiter’s bow and pelvic tilt tests, correlated strongly with patient-reported physical function and were practical in outpatient settings. Postoperative HRQoL scores were comparable to those of the general Finnish female population, regardless of the surgical method^16^. Patients with more severe preoperative urinary symptoms experienced greater symptomatic improvement. The limited benefit observed from physical training in this cohort likely reflects the participants’ substantial pre-enrolment training efforts. These results support existing evidence that surgical intervention is a viable secondary option when conservative measures prove insufficient^10,25^.

RD frequently coexists with hernias^8^, for which mesh augmentation is commonly employed to enhance repair durability and reduce recurrence. Mesh is recommended for umbilical and midline hernias, particularly when the IRD is >50 mm^24,26^. While some suggest suture repair for ventral hernias <30 mm with concurrent RD, this contrasts with EHS guidelines, which advise mesh reinforcement for defects >10 mm^1,27,28^. Numerous endoscopic onlay repair (ENDOR) techniques exist for ventral hernia repair with RD plication, though they often neglect excess skin resulting from fascial tightening^29^. A systematic review by ElHawary *et al*.^5^ reported low recurrence and complication rates for both open and laparoscopic approaches. Only a limited number of RCTs have compared mesh and suture techniques. Emanuelsson *et al*.^6^ found no significant differences in abdominal wall stability or complication rate between Stoppa-style mesh-augmented and suture-only techniques. Another RCT compared Duramesh (Mesh Suture, Inc., Chicago, IL, USA) with suture plication and reported no significant differences in infection, seroma, haematoma, wound dehiscence, fistula rates, pain, or BODY-Q outcomes^30^.

This study has several limitations. First, the PSUM group had a significantly wider baseline IRD, which may partially explain the greater postoperative reduction observed. Second, a major limitation of this study was premature termination due to COVID-19-related lockdowns resulting in not achieving the planned patient enrolment. This makes the trial underpowered to detect small differences in relapse rates, especially as it was designed to identify a 30% *versus* 5% difference, while the observed relapse rates were substantially lower. A third major limitation is the absence of a non-surgical control group. The fourth major limitation is the 1 year follow-up, as it does not capture long-term durability; extended follow-up is planned. Although aesthetic outcomes were not a primary endpoint, they may have contributed to the observed HRQoL improvements.

Plication remains an effective technique for RD repair either with or without a mesh, but the study was limited by both a small number of patients and a lower than anticipated overall RD recurrence rate. However, for repairs requiring additional reinforcement—such as for patients with a poor-quality linea alba or concomitant midline hernias, the PSUM technique may offer a valuable alternative. This study contributes to the growing body of literature supporting surgical treatment for symptomatic RD, demonstrating improvements in both core function and patient QoL.

## Supplementary Material

znaf231_Supplementary_Data

## Data Availability

Deidentified data may be shared upon reasonable request.
