# Supplementary material for: Rectus diastasis repair with and without mesh at 1 year: randomized clinical trial
Source: Br J Surg. 2025 Nov 25;112(11):znaf231. doi: 10.1093/bjs/znaf231 (PMC12646141; doi:10.1093/bjs/znaf231)
Supplement: znaf231_Supplementary_Data [file znaf231_supplementary_data.zip › Study protocol.docx]

**Study Overview**

**Brief Summary**

This study is a randomized controlled trial comparing two ADR repair methods: nylon suturing and nylon suture with mesh enforcement. The ADR correction is performed simultaneously with abdominoplasty/ modified skin reduction abdominoplasty.

**Detailed Description**

Abdominal diastasis recti (ADR) persists after pregnancies in one third of women. Traditionally plain ADR has been managed conservatively. There is some evidence that ADR reduces abdominal integrity and functional strength, contributing to pelvic instability and back pain. However, patients are referred to a surgeon mainly because of some other primary concern and ADR is an additional condition: in the case of excess skin-subcutis, the person is referred to a plastic and reconstructive surgeon for abdominoplasty and in the case of midline hernia, to a general surgeon.

In combination with abdominoplasty the plication of the superficial aponeurosis of recti muscles is the most commonly used reconstructive technique. There is a wide variety of different plication procedures available. Convincing data of the long-term results of ADR repair are lacking especially when ADR is severe. Some studies have reported large recurrence rates. Polypropylene mesh repair is an evidence-based technique to ensure a strong and reliable abdominal wall repair in ventral hernias or in high risk laparotomy wounds. Large retromuscular or intraperitoneal meshes have been used also in ARD repair.

This study reports a novel surgical technique aimed at reliable and mini-invasive open repair of ADR with or without midline hernia combined by abdominoplasty for symptomatic ADR patients. In RmB (roll mesh in between) method the investigators bury a narrow piece of self-gripping mesh inside the plicated linea alba to give tensile strength to plication. Patients are randomized to a suture plication group or RmB group.

Outcome evaluation is performed by clinical examination with video recorded movement control tests and with structured questionnaires for Quality of Life (RAND36) and for low back pain (LBP) (Oswestry 2.0). Evaluation is done three times: when recruiting the patient, after a conservative 3-6 months therapy with written instructions and one year after the intervention. Complications and recurrences are recorded as well.

Outcomes The effect of ADR repair on LBP and movement control problems Patient satisfaction and complications of ADR repair after the two techniques

**Official Title**

Operative Correction of Abdominal Rectus Diastasis (ARD): the Effect on Low Back Pain and Movement Control. A Randomized, Prospective Trial Comparing Novel, Mini-invasive Mesh Repair to Plication

**Conditions**

Diastasis Recti

**Intervention / Treatment**

- Procedure: Suture repair
- Procedure: Rolled mesh repair

**Other Study ID Numbers**

- HUS/26/2018

**Study Start (Actual)**

2018-04-01

**Primary Completion (Estimated)**

2019-12-31

**Study Completion (Estimated)**

2021-12-31

**Enrollment (Estimated)**

100

**Study Type**

Interventional

**Contacts and Locations**

This section provides contact details for people who can answer questions about joining this study, and information on where this study is taking place.

To learn more, please see the [Contacts and Locations section in How to Read a Study Record](https://clinicaltrials.gov/study-basics/how-to-read-study-record#contacts-and-locations).

**Study Contact**

**Name:**Jaana Vironen

**Phone Number:**+358504422892

**Email:**[jaana.vironen@hus.fi](mailto:jaana.vironen@hus.fi?subject=NCT03509376,%20HUS/26/2018,%20Operative%20Correction%20of%20Rectus%20Muscle%20Diastasis%20(ARD):%20the%20Effect%20on%20Low%20Back%20Pain%20and%20Movement%20Control)

**Study Contact Backup**

**Name:**Reetta Tuominen

**Email:**[reetta.tuominen@hus.fi](mailto:reetta.tuominen@hus.fi?subject=NCT03509376,%20HUS/26/2018,%20Operative%20Correction%20of%20Rectus%20Muscle%20Diastasis%20(ARD):%20the%20Effect%20on%20Low%20Back%20Pain%20and%20Movement%20Control)

This study has 2 locations

**Finland**

**[Espoo, Finland, 02480](https://clinicaltrials.gov/)**

**[Recruiting](https://clinicaltrials.gov/)**

[HUCH Jorvi Hospital, department of Surgery](https://clinicaltrials.gov/)

[Contact:](https://clinicaltrials.gov/)

[Jaana Vironen, MD PhD
+358 50 4422892 jaana.vironen@hus.fi](https://clinicaltrials.gov/)

**Participation Criteria**

Researchers look for people who fit a certain description, called eligibility criteria. Some examples of these criteria are a person's general health condition or prior treatments.

For general information about clinical research, read [Learn About Studies](https://clinicaltrials.gov/study-basics/learn-about-studies).

**Eligibility Criteria**

**Description**

Inclusion Criteria:

- Symptomatic diastasis recti (> 3 cm) after pregnancies, with or without a midline hernia

Exclusion Criteria:

- BMI > 28,
- smoking
- less than a year since the previous pregnancy or still breast feeding
- planning further pregnancies

[Show less](https://clinicaltrials.gov/)

**Ages Eligible for Study**

18 Years to 60 Years (Adult )

**Sexes Eligible for Study**

Female

**Accepts Healthy Volunteers**

No

**Study Plan**


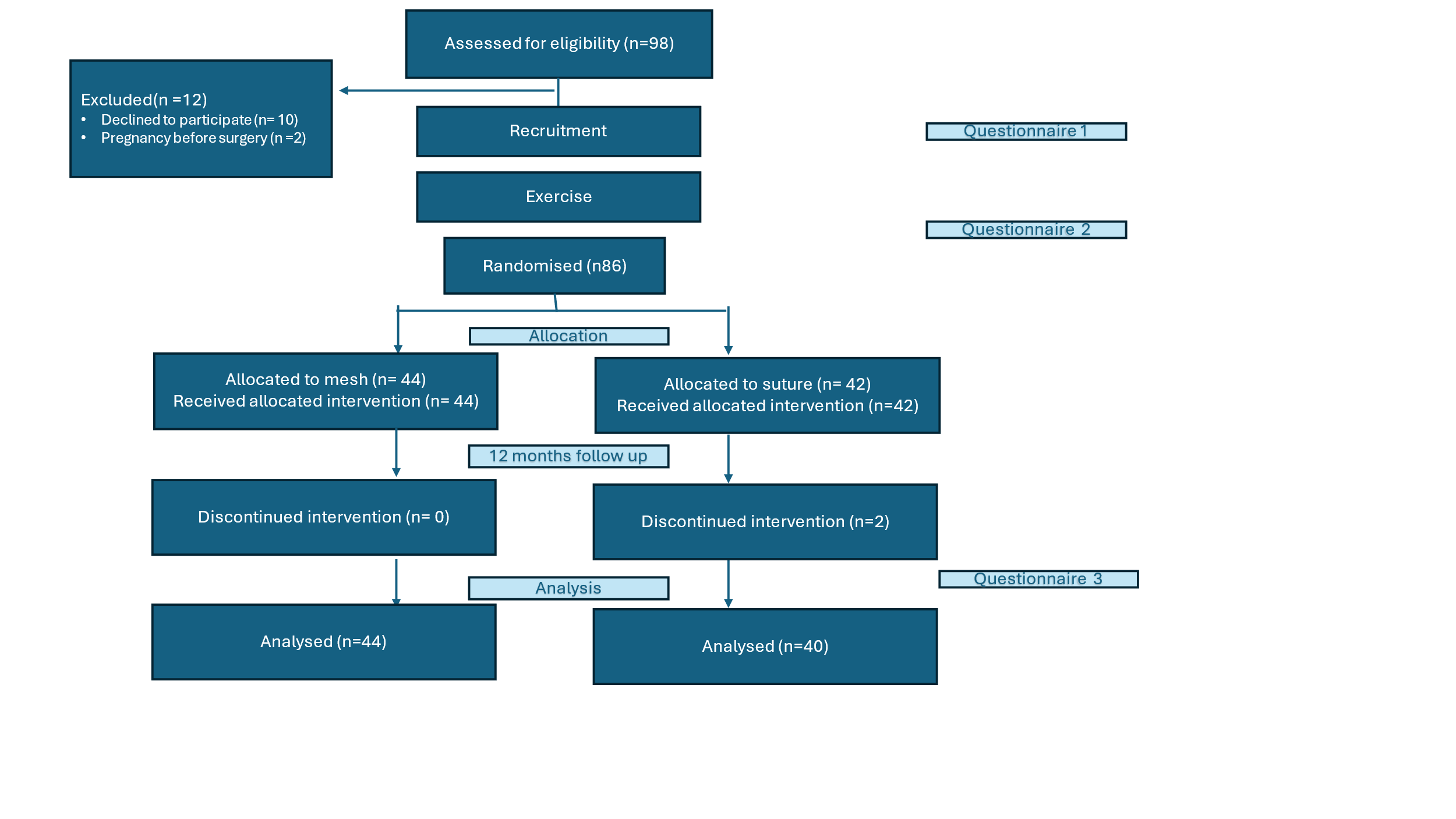


**Design Details**

**Primary Purpose  :**Treatment

**Allocation  :**Randomized

**Interventional Model  :**Parallel Assignment

**Interventional Model Description:**Patients are randomised to a suture repair or suture with mesh repair

**Masking  :**Single (Outcomes Assessor)

**Masking Description:**The physiotherapist doing the clinical examinations at different phases of the study

**Arms and Interventions**

| **Participant Group/Arm** | **Intervention/Treatment** |
| --- | --- |
| Experimental: Suture repair  Diastasis recti is repaired using nylon suture for the plication | Procedure: Suture repair   - The diastasis is repaired with continous nylon suture |
| Experimental: Rolled mesh repair  Diastasis recti is repaired with self gripping mesh to reinforce the suture line | Procedure: Rolled mesh repair   - Continuous nylon suture is done over a narrow strip of self gripping Rolled mesh to repair the diastasis |

What is the study measuring?

**Primary Outcome Measures**

| **Outcome Measure** | **Measure Description** | **Time Frame** |
| --- | --- | --- |
| Recurrence | Number of symptomatic, recurrent diastasis > 3 cm | At one year |

**Secondary Outcome Measures**

| **Outcome Measure** | **Measure Description** | **Time Frame** |
| --- | --- | --- |
| Low back Pain | ODI | at 1 year |
| Quality of life | RAND 36 questionnaire | at one year |

**Collaborators and Investigators**

This is where you will find people and organizations involved with this study.

**Sponsor**

**Helsinki University Central Hospital**

**Collaborators**

- Oulu University Hospital (Oulu did not participate due to lack of operatibve resources)

**Investigators**

- Principal Investigator:Tiina Jahkola,Helsinki University Central Hospital
- Principal Investigator:Jaana Vironen,Helsinki University Central Hospital

**Study Record Dates**

These dates track the progress of study record and summary results submissions to ClinicalTrials.gov. Study records and reported results are reviewed by the National Library of Medicine (NLM) to make sure they meet specific quality control standards before being posted on the public website.

**Study Registration Dates**

**First Submitted**

2018-04-11

**First Submitted that Met QC Criteria**

2018-04-25

**First Posted**

2018-04-26

**Study Record Updates**

**Last Update Submitted that met QC Criteria**

2018-04-25

**Last Update Posted**

2018-04-26

**Last Verified**

2018-04
