# Supplementary material for: Rectus diastasis repair with and without mesh at 1 year: randomized clinical trial
Source: Br J Surg. 2025 Nov 25;112(11):znaf231. doi: 10.1093/bjs/znaf231 (PMC12646141; doi:10.1093/bjs/znaf231)
Supplement: znaf231_Supplementary_Data [file znaf231_supplementary_data.zip › Supplementary Tables_proof read.docx]

Supplementary Tables

Table S1

| HRQoL scores between the mesh and suture groups | | |  |  |  |  |  |  |  |
| --- | --- | --- | --- | --- | --- | --- | --- | --- | --- |
|  |  |  |  |  |  |  |  |  |  |
| RAND-36 domains | Before surgery or work out |  |  | After work out, before surgery |  |  | After surgery |  | p-value |
|  | Mesh | Suture | p-value | Mesh | Suture | p-value | Mesh | Suture |  |
| Physical functioning , mean | 80.5 | 76.8 | 0.347 | 82.2 | 76.1 | 0.241 | 96.0 | 93.4 | 0.022 |
| Bodily pain | 63.0 | 56.7 | 0.177 | 63.8 | 58.8 | 0.411 | 88.6 | 82.4 | 0.219 |
| General health | 67.3 | 60.0 | 0.094 | 68.8 | 60.4 | 0.052 | 80.2 | 73.8 | 0.236 |
| Physical role functioning | 69.8 | 64.7 | 0.481 | 63.2 | 63.2 | 0.717 | 94.3 | 88.2 | 0.140 |
| Social functioning | 75.0 | 76.3 | 0.775 | 81.1 | 76.4 | 0.442 | 95.5 | 88.2 | 0.006 |
| Vitality | 45.9 | 37.4 | 0.018 | 44.0 | 42.2 | 0.389 | 54.4 | 47.9 | 0.079 |
| Mental health | 72.9 | 68.1 | 0.280 | 71.4 | 70.0 | 0.741 | 78.1 | 71.1 | 0.088 |
| Emotional role functioning | 79.8 | 76.3 | 0.412 | 75.5 | 80.6 | 0.333 | 85.6 | 76.3 | 0.232 |

Table S2

| Mesh and suture groups pre- and postoperatively in ODI scores | | | | |  |  |  |  |  |
| --- | --- | --- | --- | --- | --- | --- | --- | --- | --- |
|  |  |  |  |  |  |  |  |  |  |
|  | Before surgery or work out |  |  | After work out, before surgery |  |  | After surgery |  |  |
|  | Mesh | Suture | p-value | Mesh | Suture | p-value | Mesh | Suture | p-value |
| ODI, mean % | 13.2 | 18.4 | 0.044 | 12.9 | 17.5 | 0.175 | 3.5 | 4.6 | 0.422 |
| INCO, mean | 1.2 | 1.4 | 0.357 | 1.1 | 1.1 | 0.373 | 0.7 | 1.0 | 0.238 |
|  |  |  |  |  |  |  |  |  |  |
| ODI Oswestry 2.0 Back Pain Disability Index | | | |  |  |  |  |  |  |
| INCO Incontinence score, mean | | |  |  |  |  |  |  |  |

Table S3

| Sit-Up and Motor Control Test Performance in Mesh and Suture Groups | | | | |  |  |  |  |  |  |
| --- | --- | --- | --- | --- | --- | --- | --- | --- | --- | --- |
| a) |  |  |  |  |  |  |  |  |  |  |
|  | Before surgery |  |  |  |  | After surgery |  |  |  |  |
|  | Mesh |  | Suture |  | p-value | Mesh |  | Suture |  | p-value |
|  | A | F | A | F |  | A | F | A | F |  |
| Waiters bow | 30 | 13 | 29 | 11 | 0.516 | 30 | 10 | 32 | 6 | 0.305 |
| Pelvic Tilt | 36 | 7 | 29 | 11 | 0.331 | 40 | 0 | 38 | 2 | 0.331 |
| One Leg Stance | 34 | 9 | 20 | 20 | 0.340 | 36 | 4 | 28 | 9 | 0.148 |
| Active Straight Leg | 42 | 2 | 43 | 1 | 0.317 | 32 | 2 | 38 | 0 | 0.610 |
|  |  |  |  |  |  |  |  |  |  |  |
| Accepted performance | A |  |  |  |  |  |  |  |  |  |
| Not accepted performance | F |  |  |  |  |  |  |  |  |  |
|  |  |  |  |  |  |  |  |  |  |  |
|  |  |  |  |  |  |  |  |  |  |  |
|  |  |  |  |  |  |  |  |  |  |  |
| b) | Before surgery |  |  | After surgery | |  |  |  |  |  |
|  | Mesh | Suture | p-value | Mesh | Suture | p-value |  |  |  |  |
| Sit Up, mean of repetitions | 1.0 | 1.1 | 0.821 | 4.1 | 3.2 | 0.421 |  |  |  |  |
|  |  |  |  |  |  |  |  |  |  |  |
