# Supplementary material for: Rectus diastasis repair with and without mesh at 1 year: randomized clinical trial
Source: Br J Surg. 2025 Nov 25;112(11):znaf231. doi: 10.1093/bjs/znaf231 (PMC12646141; doi:10.1093/bjs/znaf231)
Supplement: znaf231_Supplementary_Data [file znaf231_supplementary_data.zip › Supplementary_Material.docx]

**Comparative Outcomes of Rectus Diastasis Repair with and without Mesh: A Randomized Clinical Trial**

**Authors**

Reetta Tuominen, MD, PhD^1^

Julia Saxen, MD^1^

Tiina Jahkola, MD, PhD^1^

Jani Mikkonen, DC, PhD^2^

Jari Arokoski, MD, professor ^3^

Hannu Luomajoki, Dipl. PT OM, professor ^4^

Jaana Vironen, MD, PhD ^5^

^1^ Department of Plastic Surgery, Helsinki University Hospital and University of Helsinki, Finland

^2^ Department of Surgery (incl. Physiatry), Institute of Clinical Medicine, University of Eastern Finland, Kuopio, Finland, Helsinki, Finland

^3^ Department of Physical and Rehabilitation Medicine, Helsinki University Hospital and University of Helsinki, Finland

^4^ ZHAW School of Health Professions, Zurich University of Applied Sciences, Switzerland

^5^ Abdominal Center, Helsinki University Hospital and University of Helsinki, Finland

**Corresponding author**

Reetta Tuominen, Department of Plastic Surgery, Helsinki University Hospital and University of Helsinki, PL 266, 00029 HUS

Finland

ORCID 0000-0003-0025-4388;

**Supplementary Materials - Index**

| **Supplementary Methods** |  |
| --- | --- |
| CONSORT check list | *page 3* |
|  |  |
| **Supplementary Results** |  |
| - |  |
|  |  |
| **Supplementary Appendixes** |  |
| - |  |
|  |  |
| **Supplementary Figures and Tables** |  |
| Figure S1 PSUM mesh technique | *page 3* |
| Table S1 HRQoL scores between the mesh and suture groups | *page 4* |
| Table S2 Mesh and suture groups pre- and postoperatively in ODI scores | *page 4* |
| Table S3 Sit-Up and Motor Control Test Performance in Mesh and Suture Groups | *page 5* |
|  |  |
|  |  |
| - |  |
|  |  |
|  |  |
|  |  |

**Figure S1**

**
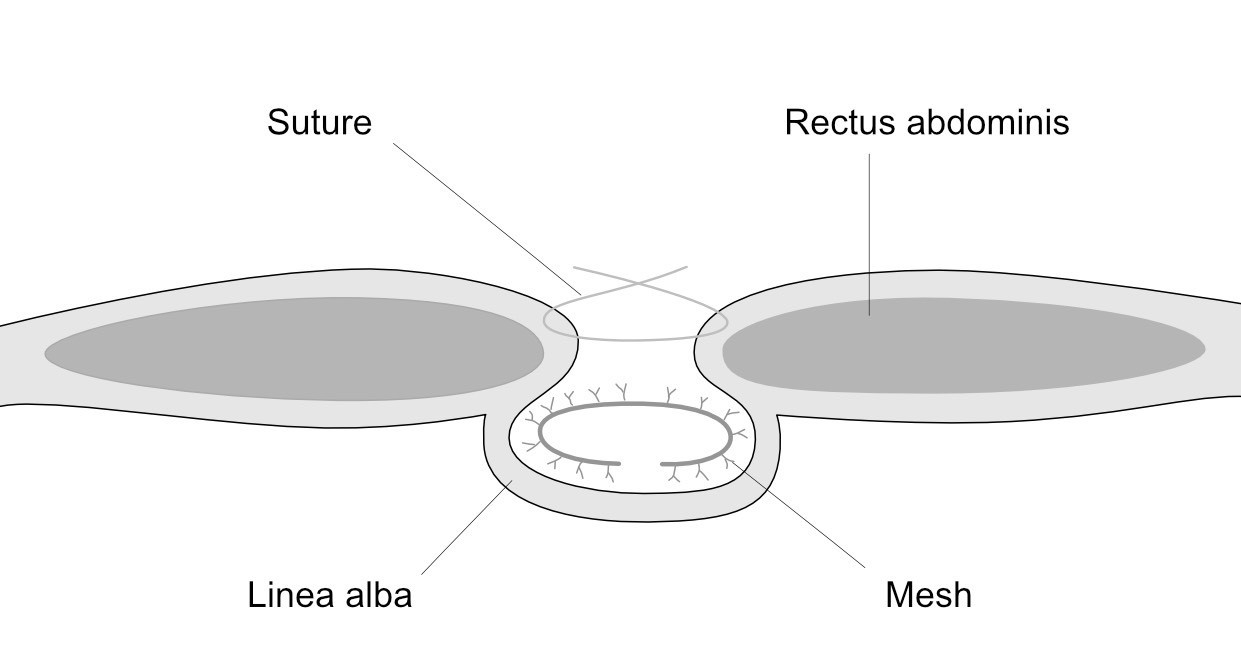
**

**Supplementary Tables**

Supplementary Tables

Table S1

| HRQoL scores between the mesh and suture groups | | |  |  |  |  |  |  |  |
| --- | --- | --- | --- | --- | --- | --- | --- | --- | --- |
|  |  |  |  |  |  |  |  |  |  |
| RAND-36 domains | Before surgery or work out |  |  | After work out, before surgery |  |  | After surgery |  | p-value |
|  | Mesh | Suture | p-value | Mesh | Suture | p-value | Mesh | Suture |  |
| Physical functioning , mean | 80.5 | 76.8 | 0.3 | 82.2 | 76.1 | 0.2 | 96.0 | 93.4 | <0.05 |
| Bodily pain | 63.0 | 56.7 | 0.2 | 63.8 | 58.8 | 0.4 | 88.6 | 82.4 | 0.2 |
| General health | 67.3 | 60.0 | 0.09 | 68.8 | 60.4 | 0.06 | 80.2 | 73.8 | 0.2 |
| Physical role functioning | 69.8 | 64.7 | 0.5 | 63.2 | 63.2 | 0.7 | 94.3 | 88.2 | 0.1 |
| Social functioning | 75.0 | 76.3 | 0.8 | 81.1 | 76.4 | 0.4 | 95.5 | 88.2 | <0.05 |
| Vitality | 45.9 | 37.4 | <0.05 | 44.0 | 42.2 | 0.4 | 54.4 | 47.9 | 0.08 |
| Mental health | 72.9 | 68.1 | 0.3 | 71.4 | 70.0 | 0.8 | 78.1 | 71.1 | 0.09 |
| Emotional role functioning | 79.8 | 76.3 | 0.4 | 75.5 | 80.6 | 0.3 | 85.6 | 76.3 | 0.2 |

Table S2

| Mesh and suture groups pre- and postoperatively in ODI scores | | | | |  |  |  |  |  |
| --- | --- | --- | --- | --- | --- | --- | --- | --- | --- |
|  |  |  |  |  |  |  |  |  |  |
|  | Before surgery or work out |  |  | After work out, before surgery |  |  | After surgery |  |  |
|  | Mesh | Suture | p-value | Mesh | Suture | p-value | Mesh | Suture | p-value |
| ODI, mean % | 13.2 | 18.4 | 0.06 | 12.9 | 17.5 | 0.07 | 3.5 | 4.6 | 0.6 |
| INCO, mean | 1.2 | 1.4 | 0.5 | 1.1 | 1.1 | 0.8 | 0.7 | 1.0 | 0.4 |
|  |  |  |  |  |  |  |  |  |  |
| ODI Oswestry 2.0 Back Pain Disability Index | | | |  |  |  |  |  |  |
| INCO Incontinence score, mean | | |  |  |  |  |  |  |  |

Table S3

| Sit-Up and Motor Control Test Performance in Mesh and Suture Groups | | | | |  |  |  |  |  |  |
| --- | --- | --- | --- | --- | --- | --- | --- | --- | --- | --- |
| a) |  |  |  |  |  |  |  |  |  |  |
|  | Before surgery |  |  |  |  | After surgery |  |  |  |  |
|  | Mesh |  | Suture |  | p-value | Mesh |  | Suture |  | p-value |
|  | A | F | A | F |  | A | F | A | F |  |
| Waiters bow | 30 | 13 | 29 | 11 | 0.97 | 30 | 10 | 32 | 6 | 0.47 |
| Pelvic Tilt | 36 | 7 | 29 | 11 | 0.33 | 40 | 0 | 38 | 2 | 0.49 |
| One Leg Stance | 34 | 9 | 20 | 20 | 0.06 | 36 | 4 | 28 | 9 | 0.10 |
| Active Straight Leg | 42 | 2 | 43 | 1 | 0.99 | 32 | 2 | 38 | 0 | 0.49 |
|  |  |  |  |  |  |  |  |  |  |  |
| Accepted performance | A |  |  |  |  |  |  |  |  |  |
| Not accepted performance | F |  |  |  |  |  |  |  |  |  |
|  |  |  |  |  |  |  |  |  |  |  |
|  |  |  |  |  |  |  |  |  |  |  |
|  |  |  |  |  |  |  |  |  |  |  |
| b) | Before surgery |  |  | After surgery | |  |  |  |  |  |
|  | Mesh | Suture | p-value | Mesh | Suture | p-value |  |  |  |  |
| Sit Up, mean of repetitions | 1.0 | 1.1 | 0.82 | 4.1 | 3.2 | 0.42 |  |  |  |  |
|  |  |  |  |  |  |  |  |  |  |  |
